# Pandemic (H1N1) 2009 in Neonates, Japan

**DOI:** 10.3201/eid1709.101803

**Published:** 2011-09

**Authors:** Naoto Takahashi, Hiroyuki Kitajima, Satoshi Kusuda, Ichiro Morioka, Kazuo Itabashi

**Affiliations:** Author affiliations: Jichi Medical University School of Medicine, Shimotsuke, Japan (N. Takahashi);; Osaka Prefectural Medical Center for Perinatal Medicine and Child Health, Osaka, Japan (H. Kitajima);; Tokyo Women’s Medical University, Tokyo, Japan (S. Kusuda);; Kobe University Graduate School of Medicine, Kobe, Japan (I. Morioka);; Showa University School of Medicine, Tokyo (K. Itabashi)

**Keywords:** pandemic (H1N1) 2009, neonates, preterm labor, influenza, viruses, Japan, letter

**To the Editor:** In 2009 in Japan, a medical response to pandemic (H1N1) 2009 infection in neonates was proposed by the Japan Pediatric Society (JPS) (*1*). Few such cases have been reported (*2**–**7*). Because the effects of pandemic (H1N1) 2009 in neonates are unknown, the JPS Committee of Neonatal Medicine conducted a nationwide survey during 2009. Surveys were mailed to neonatal care units in 522 facilities certified by JPS as teaching hospitals, which included almost all tertiary neonatal intensive care units in Japan. The survey asked whether during April 2009–March 2010 any neonates had been born to mothers with pandemic (H1N1) 2009 onset from 7 days before delivery until obstetric discharge and whether pandemic (H1N1) 2009 developed in any neonates <28 days of age before hospital discharge. The study was approved by the Bioethics Committee and Board of Directors of JPS.

Of the 522 facilities, 327 (62.6%) responded. During the period in question, 52,774 neonates had been hospitalized for any cause except routine care. Pandemic (H1N1) 2009 infection of the mother was reported by 47 (16.1%) facilities. From the 37 of these facilities, detailed information was available for ≈42 mothers with pandemic (H1N1) 2009 infection who gave birth to 43 neonates (29 full-term and 14 preterm births). Of these 42 mothers, a diagnosis of influenza A was made by rapid influenza diagnostic kit for 33 (78.6%) and for pandemic (H1N1) 2009 by reverse transcription PCR (RT-PCR) for 5 (11.9%). Only 1 case of pandemic (H1N1) 2009 in a mother was reported in May 2009, when influenza subtype H3 was dominant in Japan. This infection was confirmed by RT-PCR, and the mother was included in the study.

During the study period, except April and May 2009, almost all influenza A infections were caused by pandemic (H1N1) 2009 virus. Delivery on the day after symptom onset was most frequent (14 [32.6%] births), followed by delivery on the same day as symptom onset (8 [18.6%] births). A similar trend was observed for preterm births. Of the 42 mothers, 40 (95.2%) received antiviral medications. Mixed feeding of breast and formula milk was most common, and 8 neonates were breast-fed only.

Among the 43 neonates, pandemic (H1N1) 2009 infection developed in only 1 (male, gestational age 37 weeks, birthweight 2,665 g). His mother had high fever, and pandemic (H1N1) 2009 was diagnosed by RT-PCR; she received oseltamivir and delivered her son 2 days after illness onset. The neonate became lethargic at 4 days of age, and pandemic (H1N1) 2009 infection was confirmed by a rapid-antigen detection kit. The neonate received oseltamivir and recovered the next day. He received oseltamivir for 5 days and was discharged at 12 days of age with no subsequent medical problems. Because onset occurred 4 days after birth, the possibility of horizontal infection from the mother cannot be excluded. Except for this 1 neonate, prophylactic antiviral drugs were not given to the other 42 neonates, none of whom became infected.

With respect to nosocomial pandemic (H1N1) 2009 infection in hospital wards caring for neonates, no cases of onset within 28 days after birth were reported. However, pandemic (H1N1) 2009 infection before discharge but after 28 days of age was reported for 6 neonates. These diagnoses were made by rapid diagnostic kit, specific RT-PCR, or both. Of these 6 neonates, 1 was born at 29 weeks of gestation and had a low birthweight (1,026 g); symptom onset at 32 days of age; and complications of respiratory distress, pneumothorax, and systemic inflammatory response syndrome. Oseltamivir was given to 5 of these 6 neonates, none had adverse effects and all 6 recovered.

Pandemic (H1N1) 2009 infection may have caused preterm labor. According to our findings, the virus does not seem to be transmitted during breast-feeding, and antiviral drugs, if given to the mothers, may not always be needed by neonates. However, because of the limitations of this observational study, these findings need further support. Dulyachai et al. confirmed vertical transmission of pandemic (H1N1) 2009 virus at 31 weeks of gestation (*5*). Jajoo and Gupta reported a 32-week-old preterm patient with pandemic (H1N1) 2009 who died of pneumonia and multiorgan failure (*6*). Maternal pandemic (H1N1) 2009 infection associated with preterm labor may adversely affect the fetus or neonate (*2**,**3**,**5**,**6*).

Our results show that pandemic (H1N1) 2009 virus infection in mothers seldom occurred in their neonates, i.e., vertical transmission was rare. This finding is consistent with the fact that few such cases have been reported (*8**,**9*). On the basis of the results of this survey, JPS published Guideline for Management of Influenza (including Pandemic [H1N1] 2009) in Neonates during the Early Postnatal Period in 2010–2011 Season (*10*).
